# Comparative *In Vitro* Activities of SMT19969, a New Antimicrobial Agent, against Clostridium difficile and 350 Gram-Positive and Gram-Negative Aerobic and Anaerobic Intestinal Flora Isolates

**DOI:** 10.1128/AAC.01136-13

**Published:** 2013-10

**Authors:** Ellie J. C. Goldstein, Diane M. Citron, Kerin L. Tyrrell, C. Vreni Merriam

**Affiliations:** R. M. Alden Research Laboratory, Culver City, California, USAa; David Geffen School of Medicine at UCLA, Los Angeles, California, USAb

## Abstract

The comparative *in vitro* activity of SMT19969, a novel, narrow-spectrum, nonabsorbable agent, was studied against 50 ribotype-defined Clostridium difficile strains, 174 Gram-positive and 136 Gram-negative intestinal anaerobes, and 40 Gram-positive aerobes. SMT19969 was one dilution more active against C. difficile isolates (MIC range, 0.125 to 0.5 μg/ml; MIC_90_, 0.25 μg/ml), including ribotype 027 strains, than fidaxomicin (range, 0.06 to 1 μg/ml; MIC_90_, 0.5 μg/ml) and two to six dilutions lower than either vancomycin or metronidazole. SMT19969 and fidaxomicin were generally less active against Gram-negative anaerobes, especially the Bacteroides fragilis group species, than vancomycin and metronidazole, suggesting that SMT19969 has a lesser impact on the normal intestinal microbiota that maintain colonization resistance. SMT19969 showed limited activity against other Gram-positive anaerobes, including Bifidobacteria species, Eggerthella lenta, Finegoldia magna, and Peptostreptococcus anaerobius, with MIC_90_s of >512, >512, 64, and 64 μg/ml, respectively. Clostridium species showed various levels of susceptibility, with C. innocuum being susceptible (MIC_90_, 1 μg/ml) and C. ramosum and C. perfringens being nonsusceptible (MIC_90_, >512 μg/ml). Activity against Lactobacillus spp. (range, 0.06 to >512 μg/ml; MIC_90_, >512 μg/ml) was comparable to that of fidaxomicin and varied by species and strain. Gram-positive aerobic cocci (Staphylococcus aureus, Enterococcus faecalis, E. faecium, and streptococci) showed high SMT19969 MIC_90_ values (128 to >512 μg/ml).

## INTRODUCTION

Clostridium difficile infection (CDI) is an increasing problem worldwide, even surpassing methicillin-resistant Staphylococcus aureus (MRSA) as the leading cause of hospital-acquired infection in community hospitals (1). In recent years, a hypervirulent strain (NAP-1; ribotype 027, REA-type BI) has emerged that causes more severe disease and higher mortality, especially in more susceptible elderly patients. A key clinical issue with contemporary antibiotic therapy for patients with CDI, which relies heavily on vancomycin or metronidazole, is recurrent disease, with up to 30% of patients experiencing at least one recurrence of CDI following initial therapy (2, 3). A new drug, fidaxomicin (4, 5), shows superiority over vancomycin with “sustained cure,” but it does not show improvement in rates of recurrent disease for patients infected with hypervirulent strains (6). Therefore, there is an unmet need for other new drugs for this serious illness.

The current theory of CDI pathogenesis (2, 7) is that the use of antimicrobials leads to unintended changes in the normal gastrointestinal microbiota that leave patients vulnerable to the effects of toxigenic C. difficile. The importance of intestinal flora is emphasized by the remarkable success of fecal microbiota transplant, which has recently been shown in a randomized control clinical trial to result in approximately a 90% success rate when treating patients suffering multiple recurrent episodes who are recalcitrant to antibiotic therapy (8). As such, one of several strategies for CDI includes the development of antimicrobial agents that are less disruptive to the normal microbiota, especially the anaerobic components.

SMT19969 [2,2′bis(4-pyridyl) 3H,3′H 5,5′bibenzimidazole] is a novel, nonabsorbable antibiotic being developed specifically for CDI that had previously been reported to have *in vitro* activity against C. difficile with a MIC_90_ of 0.125 μg/ml against a panel of 82 United Kingdom clinical isolates (9). In addition, it has a very narrow spectrum of activity, with typically >1,000-fold selectivity for C. difficile over Gram-positive and Gram-negative anaerobic and facultative members of the fecal flora, and it has been successfully tested in both the hamster and human intestinal models of CDI (9, 10, 11). To further evaluate this compound, we studied the comparative *in vitro* activity of SMT19969 against 50 ribotype-defined clinical strains of C. difficile, 174 Gram-positive and 136 Gram-negative anaerobes, and 40 Gram-positive aerobes.

## MATERIALS AND METHODS

### Bacterial strains.

The C. difficile isolates were from toxin-positive fecal samples of patients with CDI obtained in 2012 and were ribotyped as previously described (12). The other organisms were recovered from recent clinical samples (2009 to 2011) from humans, mostly from intra-abdominal sources, with some Gram-positive cocci coming from skin and soft-tissue infections. Isolates were identified by standard methods (13, 14) and stored in 20% skim milk at −70°C. They were taken from the freezer and transferred at least twice on supplemented Brucella agar for anaerobes and on Trypticase soy agar for aerobes to ensure purity and good growth. Anaerobes were incubated for 48 h and aerobes for 24 h prior to testing. Inocula were prepared by direct suspensions of cells into Brucella broth to achieve the turbidity of the 0.5 McFarland standard. Inocula for aerobes were diluted 1:10 prior to inoculating the plates.

### Drugs tested.

SMT19969 was obtained from the manufacturer (Summit Corp. PLC, Abingdon, United Kingdom) and reconstituted according to the manufacturer's instructions by suspending and diluting in dimethyl sulfoxide (DMSO). Since the higher concentrations (64 to 512 μg/ml) of SMT19969 were not soluble in water or molten agar, the final concentrations of DMSO in these plates were as high as 5%. Drug-free control plates containing 5% DMSO were included. All test organisms grew in the presence of 5% DMSO. Comparator drugs were metronidazole, vancomycin (Sigma, St. Louis, MO), and fidaxomicin (Optimer Pharmaceuticals, San Diego, CA) and were reconstituted according to the manufacturers' instructions.

For anaerobes, MICs were determined using the agar dilution method according to CLSI guidelines (M11-A8) (15) and for facultative and aerobic organisms by the agar dilution method as described in CLSI M7-A9 (16). For anaerobic organisms, supplemented Brucella agar deeps were obtained from Anaerobe Systems (Morgan Hill, CA). Defibrinated sheep blood (Hema Resources Inc., Aurora, OR) was frozen and thawed to produce laked blood. On the day of testing, laked blood and the antimicrobial agents were added to the tubes of molten agar before pouring the agar dilution plates. The strains were applied to the plates using a Steers multipronged inoculator for a final concentration of approximately 10^5^ CFU/spot. After 44 h of incubation at 36°C in the anaerobic chamber incubator, the plates were examined for growth and the MICs interpreted (7). Mueller-Hinton agar was used for the aerobic MICs. Medium for streptococci was supplemented with 5% sheep blood. Metronidazole-containing plates were incubated in the anaerobic chamber. SMT19969, metronidazole, vancomycin, and fidaxomicin were tested at concentrations of 0.03 to 512 μg/ml. Quality control strains included Bacillus fragilis ATCC 25285, C. difficile ATCC 700057, Staphylococcus aureus ATCC 29213, and Enterococcus faecalis ATCC 29212. The MIC was defined as the lowest concentration that yielded no visible growth or a marked change/reduction in growth compared to the growth controls (see photos in reference 15 for examples).

## RESULTS

Results for all organisms other than C. difficile are summarized in Table 1. MICs for various C difficile strains, categorized by ribotype, are shown in Tables 2 and 3.

**Table 1:** Comparative *in vitro* activity of SMT 19969, fidaxomicin, vancomycin, and metronidazole against anaerobic and aerobic gut bacteria

Organism (*n*) and antimicrobial agent	MIC (μg/ml)		
	Range	MIC_50_	MIC_90_
Anaerobic			
Gram-negative			
Clostridium difficile (50)			
SMT19969	0.125–0.5	0.25	0.25
Fidaxomicin	0.06–1	0.25	0.5
Vancomycin	1–8	1	4
Metronidazole	0.25–8	0.5	2
Clostridium innocuum (10)			
SMT19969	0.06–1	0.25	1
Fidaxomicin	128–512	256	256
Vancomycin	16	16	16
Metronidazole	0.5–16	1	2
Clostridium perfringens (11)			
SMT19969	1->512	>512	>512
Fidaxomicin	≥0.03–0.06	≥0.03	0.06
Vancomycin	1	1	1
Metronidazole	0.5–4	2	4
Clostridium ramosum (10)			
SMT19969	128->512	>512	>512
Fidaxomicin	>512	>512	>512
Vancomycin	4	4	4
Metronidazole	0.5–8	0.5	1
Bifidobacterium species^*a*^ (20)			
SMT19969	16->512	>512	>512
Fidaxomicin	≥0.03–0.25	0.125	0.125
Vancomycin	0.5–1	1	1
Metronidazole	2->512	32	128
Lactobacillus species^*b*^ (20)			
SMT19969	0.06->512	16	>512
Fidaxomicin	0.25->512	8	>512
Vancomycin	0.5->512	256	>512
Metronidazole	2->512	>512	>512
Eggerthella lenta (20)			
SMT19969	>512	>512	>512
Fidaxomicin	≥0.03–32	≥0.03	≥0.03
Vancomycin	1–8	2	4
Metronidazole	0.125–0.5	0.25	0.5
Other Gram-positive rod species^*c*^ (23)			
SMT19969	0.06->512	16	>512
Fidaxomicin	0.25–256	2	128
Vancomycin	0.25–4	2	4
Metronidazole	0.25->32	0.5	2
Finegoldia magna (20)			
SMT19969	0.03–512	1	64
Fidaxomicin	0.25–32	1	2
Vancomycin	0.25–1	0.5	0.5
Metronidazole	0.125–1	0.5	1
Parvimonas micra (20)			
SMT19969	≥0.015–0.5	0.125	0.25
Fidaxomicin	≥0.03–0.06	≥0.03	≥0.03
Vancomycin	0.25–1	1	1
Metronidazole	≥0.03–1	0.25	0.5
Peptostreptococcus anaerobius (20)			
SMT19969	0.125–128	64	64
Fidaxomicin	≥0.03	≥0.03	≥0.03
Vancomycin	0.5	0.5	0.5
Metronidazole	0.125–1	0.5	1
Gram-negative organisms			
Bacteroides fragilis (20)			
SMT19969	512->512	>512	>512
Fidaxomicin	>512	>512	>512
Vancomycin	32–128	64	64
Metronidazole	0.5–2	1	2
Bacteroides ovatus (10)			
SMT19969	>512	>512	>512
Fidaxomicin	>512	>512	>512
Vancomycin	64–256	128	256
Metronidazole	0.5–2	1	2
Bacteroides thetaiotaomicron (10)			
SMT19969	>512	>512	>512
Fidaxomicin	>512	>512	>512
Vancomycin	64–256	128	128
Metronidazole	0.5–2	1	2
Bacteroides vulgatus (10)			
SMT19969	128->512	>512	>512
Fidaxomicin	>512	>512	>512
Vancomycin	8–128	32	128
Metronidazole	0.5–2	0.5	1
Parabacteroides species^*d*^ (10)			
SMT19969	512->512	>512	>512
Fidaxomicin	>512	>512	>512
Vancomycin	32–128	128	128
Metronidazole	0.5–2	1	2
Fusobacterium nucleatum (10)			
SMT19969	4–64	64	64
Fidaxomicin	>512	>512	>512
Vancomycin	256->512	256	512
Metronidazole	0.06–0.25	0.125	0.25
Other Fusobacterium species^*e*^ (10)			
SMT19969	64->512	64	>512
Fidaxomicin	64->512	64	>512
Vancomycin	512->512	512	>512
Metronidazole	0.5	0.5	0.5
Porphyromonas species^*f*^ (23)			
SMT19969	≥0.015–0.5	0.25	0.5
Fidaxomicin	≥0.03–128	64	128
Vancomycin	1–64	4	16
Metronidazole	≥0.03–0.5	0.06	0.25
Prevotella species^*g*^ (23)			
SMT19969	32->512	>512	>512
Fidaxomicin	64->512	>512	>512
Vancomycin	64->512	128	512
Metronidazole	0.25–1	0.5	1
Veillonella species (20)			
SMT19969	>512	>512	>512
Fidaxomicin	16–512	128	256
Vancomycin	128->512	512	>512
Metronidazole	0.25–2	1	2
Aerobic organisms			
Staphylococcus aureus (10)			
SMT19969	>512	>512	>512
Fidaxomicin	4–16	8	16
Vancomycin	1	1	1
Metronidazole	>512	>512	>512
Enterococcus faecalis (10)			
SMT19969	128->512	>512	>512
Fidaxomicin	1–8	8	8
Vancomycin	1–4	1	4
Metronidazole	>512	>512	>512
Enterococcus faecium (10)			
SMT19969	64->512	128	128
Fidaxomicin	0.5–16	8	>128
Vancomycin	0.5–256	0.5	256
Metronidazole	256->512	>512	>512
Streptococcus species^*h*^ (10)			
SMT19969	0.5->512	128	>512
Fidaxomicin	16–128	32	128
Vancomycin	0.5–2	1	1
Metronidazole	128->512	>512	>512

a Bifidobacterium adolescentis, 7; B. angulatum, 1; B. animalis subsp. lactis, 1; B. bifidum, 1; B. breve, 2; B. catenulatum, 1; B. dentium, 1; B. longum subsp. longum, 5; B. pseudocatenulatum, 1.

b Lactobacillus acidophilus, 1; L. casei, 1; L. casei/paracasei, 2; L. crispatus, 2; L. fermentum, 3; L. gasseri, 2; L. oris, 1; L. rhamnosus, 5; L. vaginalis, 2; L. gasseri/johnsonii, 1.

c Eggerthella species, 1; Eubacterium limosum, 4; Mogibacterium timidum, 4; Pseudramibacter alactolyticus, 4; Slackia exigua, 4; Solobacterium mooreii, 5; Propionibacterium acnes, 1.

d Parabacteroides distasonis, 5; P. merdae, 5.

e Fusobacterium mortiferum, 2; F. necrophorum, 5; F varium, 3.

f Porphyromonas asaccharolytica, 15; P. gingivalis, 6; P. somerae, 2.

g Prevotella bivia, 5; P. buccae, 6; P. intermedia/nigrescens, 3; P. intermedia, 2; P. melaninogenica, 5; Alloprevotella rava, 1; Butyricimonas species, 1.

h Streptococcus pyogenes, 2; S. agalactiae, 2; S. anginosus, 2; S. constellatus, 1; S. intermedius, 1; S. pneumoniae, 2.

**Table 2:** *In vitro* activity of SMT19969 against isolates of Clostridium difficile according to grouping of ribotypes most frequently encountered in our study

Ribotype (*n*)	MIC (μg/ml)		
	Range	MIC_50_	MIC_90_
Ribotype 002 (8)			
SMT19969	0.125–0.25	0.25	
Fidaxomicin	0.06–0.25	0.25	
Vancomycin	1–2	1	
Metronidazole	0.25–0.5	0.5	
Ribotype 014 (8)			
SMT19969	0.125–0.25	0.125	
Fidaxomicin	0.06–0.5	0.25	
Vancomycin	1–2	1	
Metronidazole	0.25–0.5	0.5	
Ribotype 027 (11)			
SMT19969	0.25–0.5	0.25	0.25
Fidaxomicin	0.5–1	0.5	0.5
Vancomycin	1–8	2	4
Metronidazole	2–8	2	8
Ribotype 054 (4)			
SMT19969	0.125–0.25	0.25	
Fidaxomicin	0.125	0.125	
Vancomycin	1–2	1	
Metronidazole	0.5	0.5	

**Table 3:** MICs of other ribotypes

Ribotype	RMA no.^a^	MIC (μg/ml)			
		SMT19969	Fidaxomicin	Vancomycin	Metronidazole
005	23002	0.25	0.06	2	0.5
005	23005	0.25	0.25	2	0.5
005	23020	0.25	0.25	2	0.5
017	22993	0.25	0.25	2	0.25
019	22905	0.25	0.125	1	0.5
020	23058	0.25	0.25	1	0.5
056	22965	0.25	0.25	1	0.5
056	23009	0.25	0.25	1	0.5
056	23017	0.25	0.25	1	1
077	23019	0.25	0.25	1	1
078	22962	0.25	0.125	1	0.5
106	22953	0.25	0.5	1	0.5
106	22967	0.25	0.5	1	0.5
106	23000	0.25	0.5	1	0.5
137	22913	0.25	0.125	8	1
137	22915	0.25	0.5	8	1
153	22968	0.25	0.5	1	0.5
190	22878	0.25	0.25	8	0.5
277	23031	0.25	0.25	1	0.5

a RMA no., R. M. Alden Research Lab isolate number.

Overall, SMT19969 showed potent growth inhibition of the C. difficile isolates tested, with MIC values one or more dilutions lower than those of fidaxomicin for 42% (21/50) of strains. On a weight basis, SMT19969 MICs were equal to those of fidaxomicin for 18 strains, one dilution lower for 17 strains, two dilutions lower for 4 strains, one dilution higher for 8 strains, and two dilutions higher for three strains of C. difficile. When analyzed by ribotype, ribotype 027 MICs for SMT19969 were generally one dilution lower than those of fidaxomicin and two to six dilutions lower than those of either vancomycin or metronidazole. Both vancomycin and metronidazole had lower MICs against ribotypes 002 and 014 than ribotype 027, while SMT19969 and fidaxomicin maintained their superior activity against both ribotypes 002 and 014. There were too few isolates in the other ribotype groupings to generalize, although fidaxomicin MICs were one dilution lower than those of SMT19969 against three of the four ribotype 054 strains tested.

Both SMT19969 and fidaxomicin generally were 4- to 16-fold more active than vancomycin and 2- to 8-fold more active than metronidazole against C. difficile strains. The prior study (9) which compared SMT19969, metronidazole, and vancomycin against 82 C. difficile clinical isolates also used the agar dilution method but with Wilkins-Chalgren agar and ∼10^4^ CFU/spot inoculum, which is 1 log_10_ less than the inoculum of the CLSI procedure. For their total of 82 strains, including 10 of ribotype 027, they noted SMT19969 to have an MIC range of 0.06 to 0.125 μg/ml with a MIC_90_ of 0.125 μg/ml, which is slightly lower than our results for both general strains and ribotype 027 strains, which may be attributable to differences in media and inoculum concentration. They did note SMT19969 to be more active than either vancomycin or metronidazole in ranges similar to those of our findings.

## DISCUSSION

Louie et al. (7) speculated that “retention of components of the normal microflora,” especially B. fragilis group species, would have a lesser ecological impact and “might lower the risk of recurrent disease.” Regarding preservation of the normal anaerobic fecal flora components, both SMT19969 and fidaxomicin were inactive against all 60 Bacteroides species (B. fragilis, B. ovatus, B. thetaiotaomicron, and B. vulgatus) and Parabacteroides species isolates, with MICs of >512 μg/ml. Vickers et al. (9) tested 16 Bacteroides species isolates against SMT19969 and found that all isolates had MICs of >512 μg/ml, except for two strains of B. vulgatus with MICs of 128 μg/ml. Against other Gram-negative anaerobes, SMT19969 showed minimal activity against Veillonella, Prevotella, and Fusobacterium species other than F. nucleatum, with MIC_90_s of >512 μg/ml. F. nucleatum isolates were more susceptible to SMT19969 than other Fusobacterium species, with a MIC_90_ of 64 μg/ml (range, 4 to 64 μg/ml). As with vancomycin, Porphyromonas species were highly susceptible to SMT19969, with a MIC_90_ of 0.5 μg/ml (range, ≤0.015 to 0.5 μg/ml). Similar to fidaxomicin, the relatively poor activity against Gram-negative anaerobes suggests that SMT19969 has a lesser impact on the normal gut microbiota that maintain colonization resistance (7).

Data for Gram-positive anaerobic bacteria showed that SMT19969 was significantly more selective in its activity than fidaxomicin. Minimal growth inhibition was observed against bifidobacteria, Eggerthella lenta, Finegoldia magna, and Peptostreptococcus anaerobius, with SMT19969 MIC_90_ values of >512, >512, 64, and 64 μg/ml, respectively, compared to MIC_90_ values for fidaxomicin of 0.125, ≤0.03, 2, and ≤0.03 μg/ml. The activity of both agents tested against various lactobacilli showed comparable MIC_90_s and MIC ranges, with susceptibility varying by strain. SMT19969 MIC values against Lactobacillus paracasei and L. rhamnosus were >512 μg/ml, whereas other Lactobacillus species showed various levels of susceptibility, ranging from 0.06 to 16 μg/ml.

Against Clostridium species, growth inhibition by SMT19969 was species dependent, with MIC_90_s of >512 μg/ml recorded against C. perfringens and C. ramosum, whereas Clostridium innocuum showed high susceptibility to SMT19969 compared to fidaxomicin, with MIC_90_s of 1 and 256 μg/ml, respectively.

All the aerobes tested (S. aureus, E. faecalis, E. faecium, and streptococci) showed much higher SMT19969 MICs (128 to >512 μg/ml) than fidaxomicin (4 to 16 μg/ml for S. aureus, E. faecalis, and E. faecium and 16 to 128 μg/ml for streptococci).

Overall, SMT19969 had enhanced activity against C. difficile isolates, regardless of ribotype, compared to the other agents tested. It was generally less active than fidaxomicin, vancomycin, and metronidazole against the other Gram-positive aerobes and anaerobes tested. With this relatively narrow spectrum of activity, SMT19969 is likely to have less activity against endogenous gut organisms; thus, it shows promise as a new drug for treating CDI.
